# Preparation, characterization, and magnetic resonance imaging of Fe nanowires

**DOI:** 10.1186/s11671-023-03916-3

**Published:** 2023-10-31

**Authors:** Xiaoming Cao, Shike Hu, Hua Zheng, Aiman Mukhtar, KaiMing Wu, Liyuan Gu

**Affiliations:** 1 School of Nuclear Technology and Chemistry and Biology, Hubei University of Science and Technology, Xianning, People’s Republic of China; 2 The State Key Laboratory of Refractories and Metallurgy, Hubei Province Key Laboratory of Systems Science in Metallurgical Process, International Research Institute for Steel Technology, Collaborative Innovation Center for Advanced Steels, Wuhan University of Science and Technology, Wuhan, People’s Republic of China

**Keywords:** Template method, Nanowires, Relaxation, Biocompatibility, Contrast agents

## Abstract

A facile template method was employed to synthesize Fe nanowires of different sizes, dimensions. Comprehensive analyses were conducted to explore their morphology, structure, composition, and magnetic properties. The surface of as-prepared Fe nanowires was modified with SiO_2_ by sol–gel method to improve the dispersion of as-prepared Fe nanowires in aqueous solution. Furthermore, the relaxation properties, biocompatibility and in vivo imaging abilities of the Fe@SiO_2_ nanowires were evaluated. The study revealed that the SiO_2_-coated Fe nanowires functioned effectively as transverse relaxation time (T_2_) contrast agents (CAs). Notably, as the length of the Fe@SiO_2_ nanowires increased, their diameter decreased, leading to a higher the transverse relaxivity (r_2_) value. Our study identified that among the Fe nanowires synthesized, the Fe3@SiO_2_ nanowires, characterized by a diameter of around 30 nm and a length of approximately 500 nm, exhibited the highest r_2_ value of 59.3 mM^−1^ s^−1^. These nanowires demonstrated good biocompatibility and non-toxicity. Notably, upon conducting small animal imaging a 1.5 T with Sprague–Dawley rats, we observed a discernible negative enhancement effect in the liver. These findings indicate the promising potential of Fe@SiO_2_ nanowires as T_2_ CAs, with the possibility of tuning their size for optimized results.

## Introduction

T_2_ CAs generally interfere with the surrounding magnetic field, leading to a broader and more uneven magnetic field. As a result, adjacent hydrogen protons experience dephasing, reducing the signal intensity in T_2_-weighted magnetic resonance imaging (MRI) [1–4]. Ferrite nanoparticles, such as Fe_3_O_4_, γ-Fe_2_O_3_, and FeOOH, are commonly used as T_2_ CAs due to their excellent biocompatibility and strong magnetic moments [5–7]. Hydrogen protons become dephased under a strong magnetic field. At present, superparamagnetic iron oxide nanoparticles (SPIOs) [8–11, ] are the most prevalent T_2_ CAs. In clinical settings, the commonly used T_2_ magnetic resonance CAs include dextrose-encapsulated Fe_3_O_4_ nanoparticles (Feridex), iron carboxyglucosamine (Resist), and dextran-modified Fe_3_O_4_ (Combidex) [12–14]. Heidarshenas et al. [15] reported that the magnetic induction intensity of spherical magnetic nanoparticles increased significantly as their aspect ratio increased, even when the volume was kept constant. In a separate study, Mohapatra [16] determined that when spherical SPIO nanoparticles were elongated, the resulting Fe_3_O_4_ nanowires induced greater local magnetic field inhomogeneity compared to their spherical counterparts of the same volume. This is because the Fe_3_O_4_ nanowires possessed a larger surface area and higher susceptibility, which was conducive to accelerating the spin-dephasing process of hydrogen protons in water. This shortened the transverse relaxation time of hydrogen protons, thereby improving the T_2_ relaxation.

Previous studies on T_2_ magnetic resonance CAs have predominantly focused on spherical zero-dimensional magnetic nanoparticles; hence, there are limited reports concerning one-dimensional magnetic nanomaterials in this context. In comparison with zero-dimensional magnetic nanoparticles, one-dimensional iron-based magnetic nanomaterials exhibit superior geometric anisotropy and a larger surface area, inducing a more uneven magnetic field and increasing the likelihood of interactions between the nanomaterials and water molecules. As a consequence, they tend to circulate longer in the bloodstream and possess stronger penetration into porous tissues, such as tumor sites [17, 18]. Therefore, it is of paramount significance to study the relaxation and in vivo imaging of one-dimensional ferromagnetic Fe nanomaterials. The characteristics of these one-dimensional ferromagnetic Fe nanomaterials are directly related to their geometric dimensions, including both diameter and length [19, 20], which profoundly impacts their relaxation properties.

In the present work, the template method was employed to fabricate Fe nanowires of varying diameters and lengths on an anodic aluminum oxide (AAO) template through electrochemical deposition. The surface of the resulting nanowires was then modified with SiO_2_ to improve their stability in aqueous solution. The morphology, structure, composition, magnetic properties, and MRI performance of the as-fabricated nanowires were characterized. To assess the cytotoxicity of the Fe@SiO_2_ nanowires, we employed human cervical cancer cells (HeLa cells) and mouse embryonic fibroblasts (NIH3T3 cells) as cellular models. Moreover, the biocompatibility of the nanowires was evaluated through the analysis of biological tissue sections. Lastly, an in vivo imaging experiment on SD rats was carried out to evaluate the T_2_ contrast effect of the Fe@SiO_2_ nanowires.

## Experimental materials and methods

### Materials and instruments

Self-made AAO templates, ferrous sulfate heptahydrate (FeSO_4_·7H_2_O, 99.0 wt%), boric acid (H_3_BO_3_, 99.5 wt%), ascorbic acid (Vc, 99 wt%), absolute ethanol (C_2_H_5_OH, 99.5 wt%), phosphoric acid (H_3_PO_4_, 85 wt%), ammonia (NH_4_OH, 25 wt%), sulfuric acid (H_2_SO_4_, 70 wt%), ammonium sulfate ((NH_4_)_2_SO_4_, 99.0 wt%), sodium hydroxide (NaOH, 97.0 wt%), acetone (C_3_H_6_O, 99.5 wt%), tetraethyl orthosilicate (TEOS, 99.9 wt%), and hydrochloric acid (HCl, 37.5 wt%) were purchased from Sigma-Aldrich. Hematoxylin staining solution, NIH3T3 cells, HeLa cells, *CCK8, PBS, DMEM medium Glutamine MEM medium, and calf serum were purchased from Sinopharm Chemical Reagent Co., Ltd., and fetal bovine serum was used in this experiment. The primary equipment used in this study included an electrochemical workstation (CHI660, China), a 1.5 T MRI system (Symphony, Germany), an oscillating sample magnetometer (VSM 7407, USA), a scanning electron microscope (ZEISS-Utral55, Germany), a transmission electron microscope (JEOL-2100F, Japan), an ultra-high-resolution transmission electron microscope (JEOL ARM-200F, Japan), a Fourier-transform infrared spectrometer (IRPrestige-21, Japan), and an X-ray diffractometer (D-500, Germany).

### Method

#### Preparation of AAO templates

We utilized a high-purity aluminum sheet (99.999%) as the anode, which was prepared via cutting, ultrasonic cleaning, annealing, and polishing. Meanwhile, a graphite sheet served as the cathode, with a 0.3 mol/L mixture of sulfuric acid and oxalic acid acting as the electrolyte. The secondary oxidation method was adopted in an ice bath setting at a specified voltage. Initially, the aluminum sheet underwent oxidation for 4 h and then immersed in a mixture of H_3_PO_4_ (5 wt%) and H_2_CrO_4_ (1.8 wt%) at 60 °C for 3 h to dissolve the oxide layer. This was followed by a secondary oxidation process. Subsequently, the aluminum sheet was extracted from the anode, and its backside was smeared with a saturated copper chloride solution to dissolve any unoxidized aluminum substrate. Finally, the aluminum sheet containing transparent anodic aluminum oxide was immersed in H_3_PO_4_ (5 wt%) to expand its pores. The detailed preparation parameters for the double-pass aluminum oxide templates with different pore diameters are listed in Table 1.

**Table 1:** Preparation parameters of AAO templates

AAO	Oxidation voltage (V)	Oxidation time (h)	Reaming time (min)	Electrolyte concentration (mol/L)
1#	25	4 + 16	15	Sulfuric acid 0.3
2#	40	4 + 36	52	Oxalate acid 0.3
3#	40	4 + 48	80	Oxalate acid 0.3
4#	40	4 + 48	105	Oxalate acid 0.3

#### Preparation of Fe nanowires

The deposition of Fe nanowires was carried out in a three-electrode electrochemical system, where an AAO template coated with a gold film was used as the working electrode, a platinum sheet acted as the auxiliary electrode, and a saturated calomel electrode was employed as the reference electrode. The deposition solution prepared using distilled water comprised 80.4 g/L of FeSO_4_·7H_2_O, 25 g/L of H_3_BO_3_, and 0.1 g/L of Vc. Vc was introduced to stabilize the pH of the deposition solution, while H_3_BO_3_ acted as a buffer, maintaining the solution at a pH of approximately 3. During the preparation of Fe nanowires, Fe^2+^ ions were prone to oxidation upon exposure to air. To counteract this, a specific amount of Vc was added to the deposition solution. The preparation parameters of Fe nanowires are presented in Table 2.

**Table 2:** Preparation parameters of Fe/AAO nanowire arrays

Sample	Deposition voltage (V)	Deposition time (s)	Deposition temperature (℃)	AAO
Fe1	−1.5	18	25	1#
Fe2	−1.5	24	25	1#
Fe3	−1.5	36	25	1#
Fe4	−1.5	85	25	1#
Fe5	−1.5	36	25	2#
Fe6	−1.5	36	25	3#
Fe7	−1.5	36	25	4#

#### Water soluble treatment of nanowires

Using the conventional hydrolysis methods, silica-coated core–shell nanowires were synthesized. A certain amount of the as-fabricated nanowires was uniformly dispersed in a mixture of ethanol (140 mL) and distilled water (20 mL), resulting in a colloidal solution. To ensure even dispersion of nanowires in this solution, ultrasonic treatment was carried out for 30 min. Following this, 2 mL of concentrated ammonia was added to the solution. After mechanical stirring for ten minutes, a mixture of TEOS (1.5 mL) and ethanol (10 mL) was added dropwise to the colloidal solution, and the mixture was stirred at room temperature for 30 h. The resulting product was subsequently recovered using a magnet, washed with ethanol and distilled water several times, and lastly, dried in a vacuum oven.

#### Characterization

The morphologies of both the front and side of the AAO templates loaded with nanowires were revealed by scanning electron microscopy (SEM). Meanwhile, the morphology of the Fe nanowires was characterized using transmission electron microscopy (TEM) and high-resolution TEM (HRTEM). The elemental composition of the nanowires was detected by energy dispersive spectroscopy (EDS). The phase compositions of the AAO templates loaded with nanowires and the nanowires were analyzed using X-ray diffraction (XRD). The magnetic properties of the nanowires were examined by vibrating sample magnetometer (VSM). Further, the structural information of the coating was analyzed by Fourier-transform infrared spectrometer (FT-IR). Both T_1_ and T_2_ values were measured using a 1.5 T MRI system.

1. Morphological characterization of samples
  To examine the morphology, structure, and size of the nanowires, TEM and HRTEM were used. For this, an appropriate amount of nanowires in an ethanol solution was dispersed and sonicated briefly to disperse them fully. Thereafter, the solution was dropped onto a copper grid with 230 mesh covered with a carbon film on its surface. After allowing it to dry naturally, it was observed and imaged using TEM.
2. Composition and structural characterization of the samples
  The EDS integrated within the TEM was utilized to conduct point scanning on the sample, yielding its elemental composition.
  Next, the structure of the sample was analyzed using XRD. The scanning voltage was set at 40 kV, the tube current at 30 mA, and the X-ray excitation source was CuKα (λ = 1.541841 Å). The scanning angle range (2θ) spanned from 20° to 90°. It underwent continuous scanning at a speed of 4°/min and a sampling interval of 0.02°.
3. Magnetic characterization of samples
  The magnetization curve of the dried sample was recorded using VSM at 300 K, and the magnetic properties of the sample were calculated. The applied magnetic field strength ranged from −20 KOe to 20 KOe.
4. Characterization of surface charge properties of samples
  The surface charge properties of the nanowires were analyzed using zeta potential. For this analysis, a nanowire dispersion was prepared at a concentration of 0.5 mmol/L [Fe]. After achieving uniform ultrasonic dispersion, the pH was maintained at 7.4. The test was performed three separate times, and the average value was taken as the zeta potential value for the final sample.
5. Characterization of sample concentration
  Furthermore, ICP-AES was employed to determine the concentration of Fe in the sample. A pipette gun was used to accurately draw a specific volume (for instance, 0.5 mL) of the nanowire dispersion into a glass sample bottle. Three replicates were set, and the sample bottle was placed on a heated magnetic stirrer set to a temperature range of 200–260 °C. Once the solution evaporated completely, 1–2 mL of high-grade pure nitric acid was added, and the heating continued. After the nitric acid had fully evaporated, the process was repeated until the solution became colorless and transparent. The heating was then halted, and the solution was allowed to cool to room temperature and further diluted to a constant volume of 5 mL. Eventually, the concentration of Fe in the sample was measured.
6. Determination of relaxation time
  Initially, Fe@SiO_2_ nanowires were dispersed in deionized water, setting up the nanowires as a water dispersion sample. Using the multiple dilution method, samples with varying Fe concentration gradients were prepared (0, 0.031, 0.062, 0.125, 0.250, and 0.500 mmol/L) of Fe@SiO_2_ nanowires water dispersion which contained 1% agarose. 5 mL of each sample was taken and placed into a centrifuge tube, which was then sequentially positioned in a multifunctional test tube rack. Using a 1.5 T clinical MRI device, the relaxation times T_1_ and T_2_ of the samples, as well as their weighted images, were scanned and measured. From these measurements, their lateral T_1_ relaxivity and T_2_ relaxivity were calculated. T_1_-weighted MR images were obtained using the turbo spin echo (TSE) method with an echo time (TE) of 11 ms and various repetition times (TR) of 50, 250, 450, 650, 850, 1050, 1250, 1450, 1650, and 1850 ms. Meanwhile, T_2_-weighted MR images were acquired using the multi-spin echo (MSE) technique with a TR of 4000 ms and varying TE values of 20, 40, 60, 80, 100, 120, 140, 160, 180 s, and 200 ms.

#### Cytotoxicity test

HeLa and NIH3T3 cells were inoculated with 1 × 10^4^ pieces/well in 96-well culture plates, with each containing 100 µL of culture medium. After 24 h of cell culture, the original culture medium was replaced with a sterilized medium infused with Fe@SiO_2_ nanowires at varying Fe concentrations: 0, 20, 40, 60, 90, and 120 µg/mL. Each Fe concentration was allocated to six wells. After incubating the cells at 37 °C in a CO_2_ cell incubator (5%) for 24 h, the original culture medium in the wells was aspirated. The wells were then carefully washed twice with PBS to remove any nanowires adhering to the cell surface. Subsequently, 100 µL of fresh DMEM culture medium containing 10 µL of CCK solution was introduced to each well, and incubation was continued for an additional 4 h. Finally, the absorbance was measured at 450 nm using an enzyme-linked immunosorbent assay. The CCK8 assay was used to detect the viability changes in both HeLa and NIH3T3 cells post-culture.

#### Cell morphology observation

HeLa and NIH3T3 cells were inoculated with 1 × 10^5^ pieces/well in 24-well culture plates, with each well containing 0.5 mL of culture medium. The original culture medium was replaced with a sterilized medium containing Fe@SiO_2_ nanowires at different Fe concentrations: 0, 20, 40, 60, and 120 µg/mL. Three wells were designated for each Fe concentration. Following a 24 h incubation at 37 °C in a CO_2_ cell incubator (5%), the original culture medium from the wells was aspirated. The wells were then meticulously washed twice with PBS to remove any nanoparticles adhering to the cell surface. An optimal microscope was then employed to observe the morphological changes in cells that had been treated with the nanoparticles.

#### Preparation of biopsies and H&E (hematoxylin–eosin) staining

Six healthy SD rats, aged 6 to 8 weeks and weighing around 200 g, were divided into two groups. The control group remained untreated, while the experimental group was injected with a specific concentration of nanowires (8 mg of Fe/kg of rat weight). Afterward, the rats were euthanized using CO_2_, and their main internal organs, including the heart, liver, spleen, lung, and kidney, were dissected. A small section of these tissues was carefully cut into smaller fragments (0.5-l mm^3^), which were then preserved in a 4% polyformic acid solution, embedded in paraffin, and sliced for H&E staining. Any pathological changes in the tissues were eventually observed under optical microscopy.

#### *In vivo MRI *$\notin$

SD rats, aged 6–8 weeks and weighing roughly 200 g, were selected for in vivo 1.5 T MRI studies. A 1.5 T MRI system served as the scanning instrument, targeting the rat liver as the region of interest. The transverse and coronal positions of the rat liver were scanned using a head wire trap, and the same sequencing was utilized to acquire T_2_-weighted MR images (TR = 4803 ms and TE = 94.38 ms).

## Results and discussion

### Material characterization

**Fig. 1:**
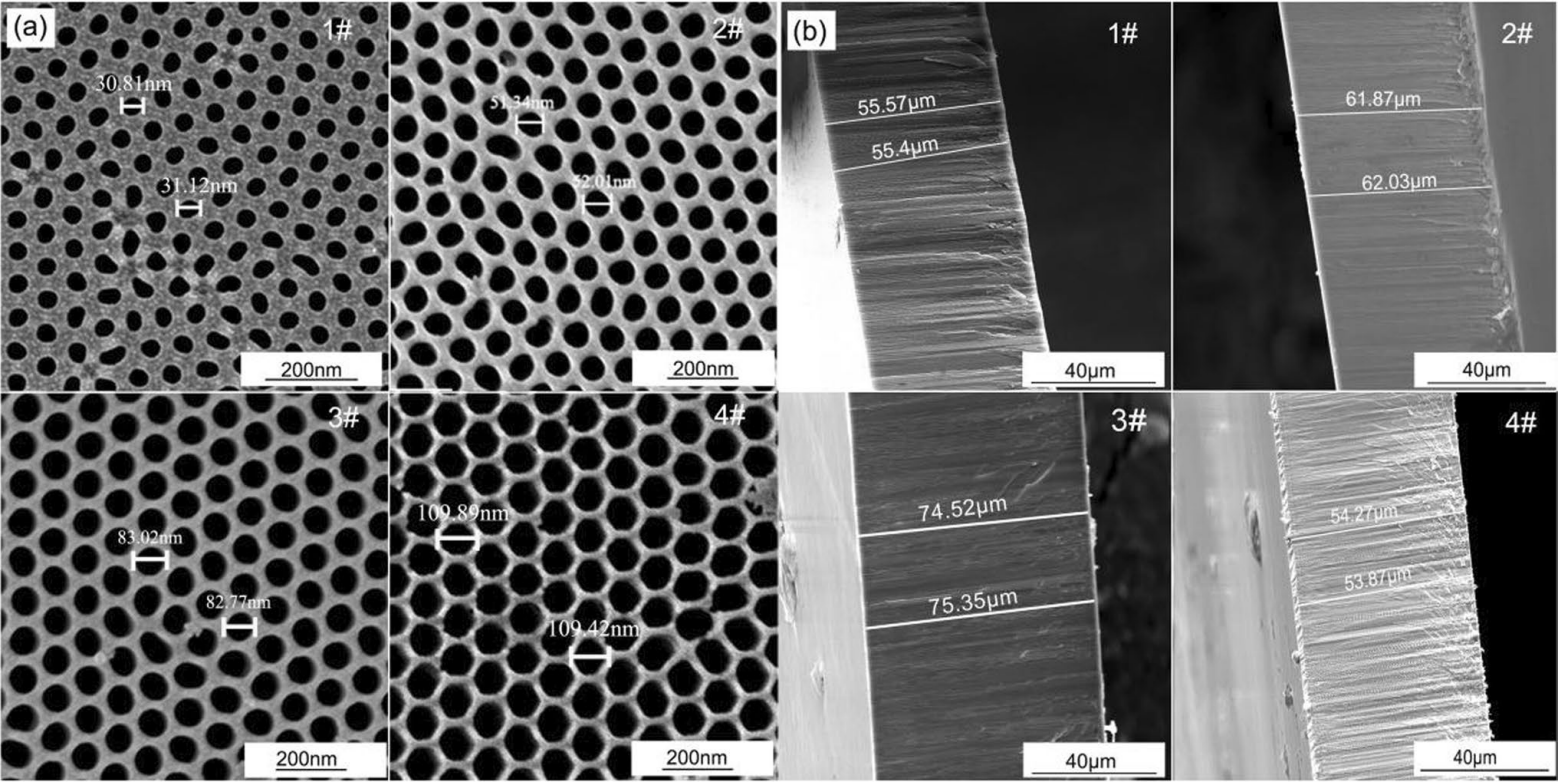
FESEM images of **a** front and **b** side of anodic aluminum oxide films prepared under different process conditions

**Fig. 2:**
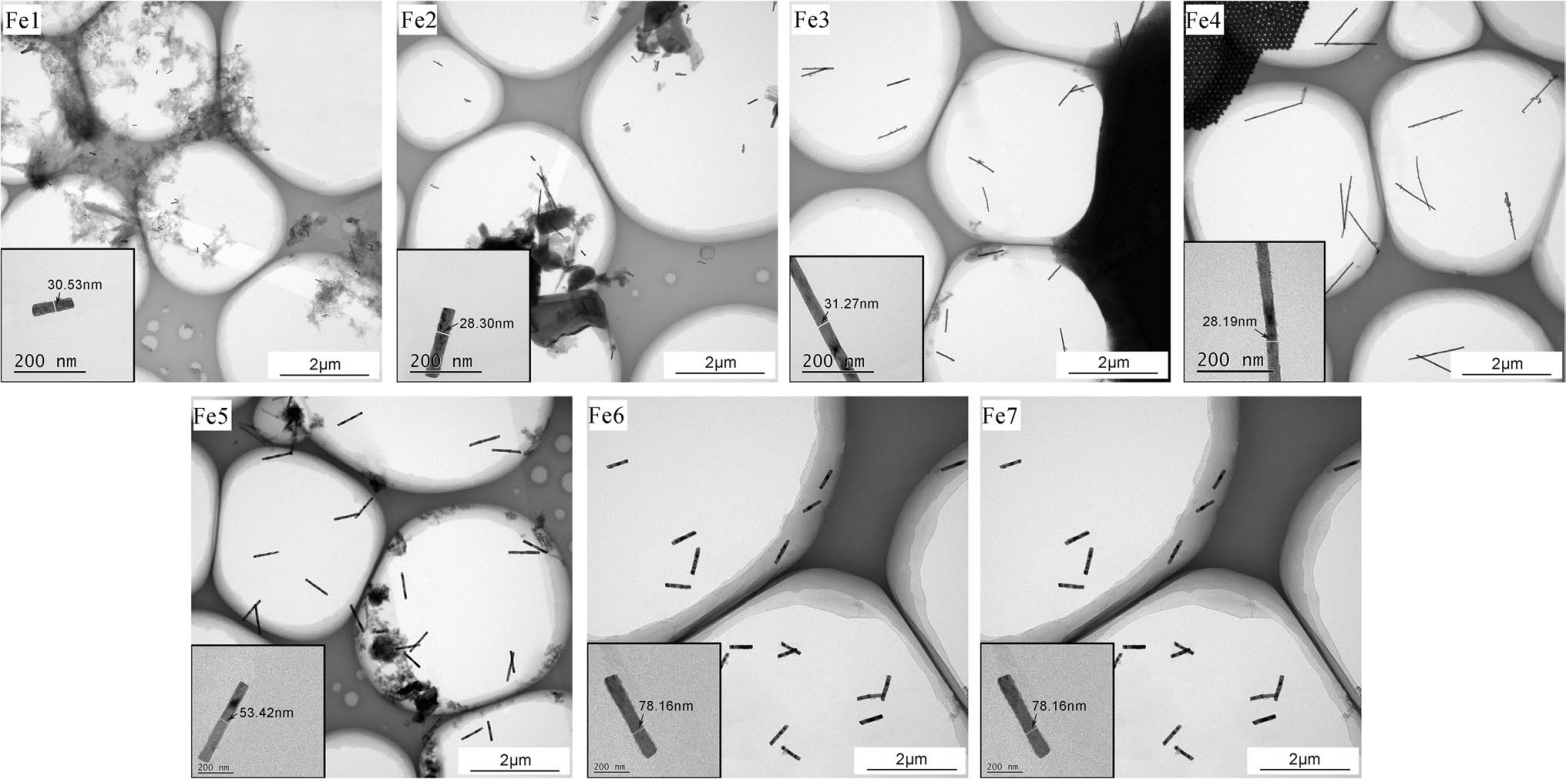
TEM images of as-deposited Fe nanowires, and the inserts are single Fe nanowire

AAO templates featuring highly ordered pores of different pore diameters (30, 50, 80, and 110 nm) are depicted in Fig. 1a, and those with film thicknesses ranging from 50–100 µm are displayed in Fig. 1b. The TEM images of the as-prepared Fe nanowires are showcased in Fig. 2. In the release of Fe nanowires from the AAO templates, ultrasonic dispersion was not used. As a result, the thickness and diameter of the Fe nanowires remained uniform, and only a limited number of them experienced breakage, given the absence of ultrasonic dispersion oscillation.

The statistical results of Fe nanowire size are presented in Table 3. The diameters of the Fe1-Fe4 nanowires measured around 30 nm, while those of the Fe5, Fe6, and Fe7 nanowires had diameters of roughly 50 nm, 80 nm, and 110 nm, respectively, aligning with the pore diameter of the AAO templates. Furthermore, as the deposition time increased, the length of the Fe nanowires grew, maintaining a growth rate of about 1 µm/min.

**Table 3:** The geometric dimensions of Fe nanowires

Fe nanowire	Diameter (nm)	Length (nm)	Aspect ratio
Fe1	29.8	104 ± 14	3
Fe2	30.5	198 ± 21	6
Fe3	29.1	521 ± 41	18
Fe4	30.9	182 ± 145	38
Fe5	52.8	595 ± 64	11
Fe6	77.5	537 ± 54	7
Fe7	108.1	514 ± 64	5

**Fig. 3:**
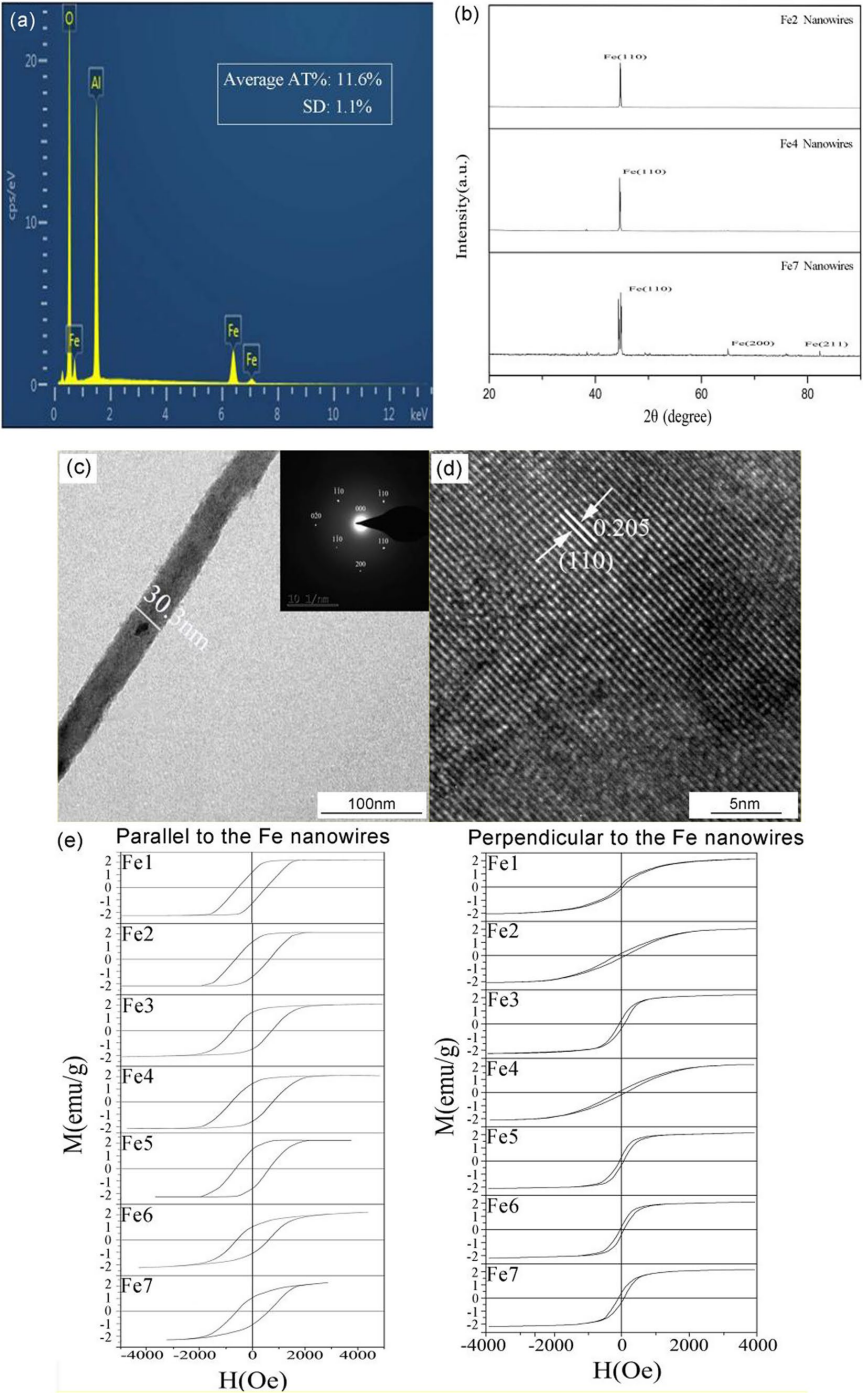
Characterization diagram of Fe nanowires, **a** EDS of Fe4/AAO nanowire arrays; **b** X-ray diffraction patterns of Fe2, Fe4, Fe7/AAO nanowire arrays; **c** a single Fe4 nanowire, illustrated with selected area electron diffraction pattern; **d** high-resolution lattice fringe phase of a single Fe4 nanowire; and **e** VSM of Fe/AAO nanowire arrays

The composition of the Fe4/AAO nanowire arrays was analyzed. As observed in Fig. 3a, the sample showed the presence of O, Fe, and Al. Notably, O and Al were generated from the AAO template, indicating the absence of any other impurities within the nanowires. Moreover, a comparison of the diffraction peak in Fig. 3b with the Fe standard card (PDF No. 06-0696) revealed that the Fe4 nanowires possessed a body-centered cubic structure.

Figure 3c presents the selected area electron diffraction pattern of a single Fe4 nanowire. The single-crystal diffraction spots along the [001] axis of the α-Fe crystal band suggested that the as-synthesized Fe4 nanowires featured a single-crystal structure. Meanwhile, Fig. 3d showcases the lattice fringe pattern of an individual Fe4 nanowire, with a lattice fringe spacing of 0.205 nm corresponding to the (110) crystal plane spacing of the Fe4 nanowires.

Later, the magnetic properties of the Fe/AAO nanowire arrays were tested using VSM. The hysteresis loops of the Fe nanowire arrays, belonging to an external magnetic field both parallel and perpendicular to them, are displayed in Fig. 3e. The associated data for these loops can be found in Tables 4 and 5.

**Table 4:** Magnetic orientation of parallel Fe nanowires

Magnetic	Fe1	Fe2	Fe3	Fe4	Fe5	Fe6	Fe7
Saturation magnetization (Ms, emu/g)	2.17	2.11	2.09	2.14	2.23	2.19	2.26
Remanence magnetization (Mr, emu/g)	1.17	1.33	1.48	1.65	1.38	1.12	0.95
Rectangle ratio (Rs = Mr/Ms)	0.54	0.63	0.71	0.77	0.62	0.51	0.42
Coercivity (Hc, Oe)	518	623	675	748	649	603	591

**Table 5:** Magnetic orientation of vertical Fe nanowires

Magnetic	Fe1	Fe2	Fe3	Fe4	Fe5	Fe6	Fe7
Saturation magnetization (Ms, emu/g)	2.1	2.07	2.18	2.09	2.15	2.13	2.17
Remanence magnetization (Mr, emu/g)	0.25	0.27	0.24	0.31	0.26	0.21	0.24
Rectangle ratio (Rs = Mr/Ms)	0.12	0.13	0.11	0.15	0.12	0.10	0.11
Coercivity (Hc, Oe)	96	110	108	116	107	105	108

When the magnetic field was applied parallel to the Fe nanowires, their Ms remained relatively low, ranging between 2.09 and 2.26 emu/g. This saturation was minimally influenced by variations in their diameter and length, as shown in Table 4. As the length of the Fe nanowires increased from 120 to 1330 nm, the Rs and Hc of the Fe1-Fe4 nanowire arrays experienced a gradual increase, with Rs rising from 0.54 to 0.77 and Hc from 518 to 748 Oe. With the increase in the diameter of the Fe nanowires from 29.1 nm to 108.1 nm, both the values of Rs and Hc showed a downward trend. Specifically, Rs values dropped from 0.71 to 0.42, while Hc values decreased from 675 to 591 Oe. This trend can be ascribed to the shape anisotropy and the multi-domain structure of the Fe nanowire arrays. Generally, the magnetization axis of nanowires is determined by the shape anisotropy of nanomaterials coupled with the magnetic dipole interaction occurring between the nanowires. Shape anisotropy directs the magnetization to align parallel to the axis of the nanowire, whereas the dipole interaction between nanowires causes the magnetization to orient perpendicular to the nanowire axis. As the aspect ratio of nanowires increased, the effect of shape anisotropy became prominent, leading the magnetization direction to align parallel to the nanowire axis.

From Table 5, it is noticed that when the applied magnetic field was perpendicular to the Fe1-Fe7 nanowire arrays, their Ms values, ranging from 2.07 to 2.18 emu/g, closely resembled the values when the applied magnetic field was parallel to the nanowire arrays. In addition, the relatively low values of Rs spanning from 0.1 to 0.15 and Hc between 96 and 116 Oe suggested that the magnetization direction of the Fe1-Fe7 nanowire arrays was parallel to the nanowire axis, primarily due to shape anisotropy.

**Fig. 4:**
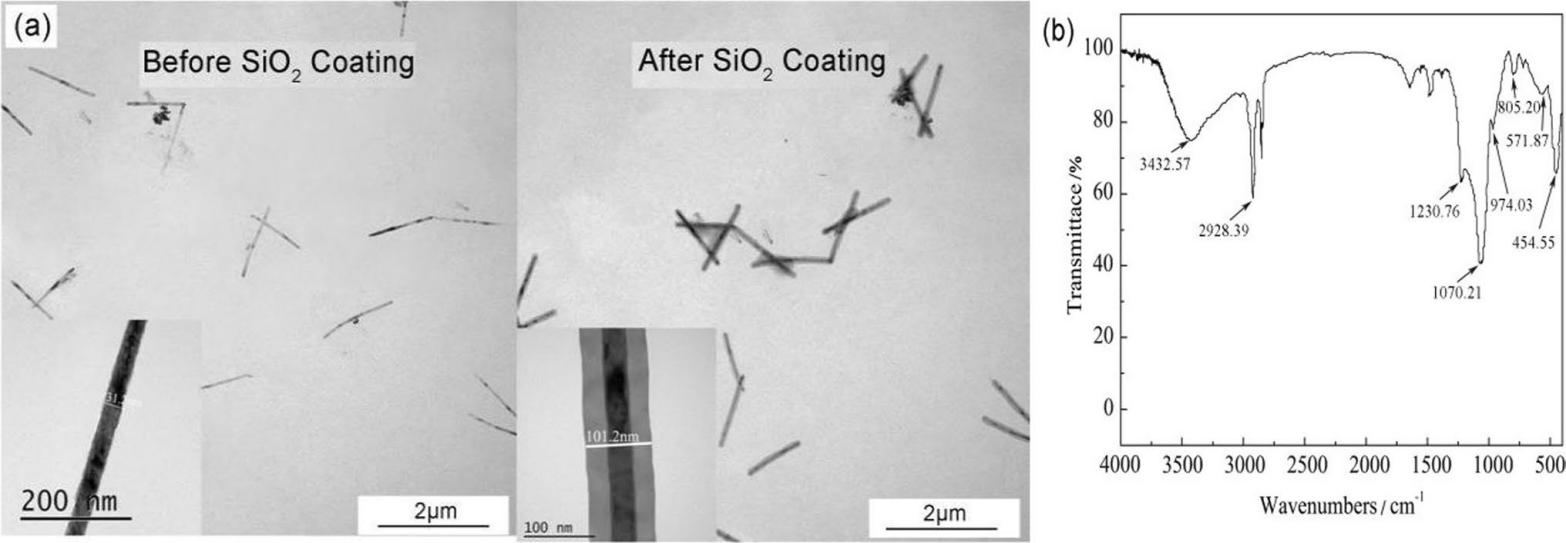
**a** TEM image of Fe4@SiO_2_ nanowires, and the inset shows enlarged an individual nanowire; **b** FT-IR spectra of Fe4@SiO_2_ nanowires

Figure 4a displays the TEM images of the Fe4 nanowires before and after SiO_2_ coating. Initially, the Fe4 nanowires exhibited a consistent linear structure with a uniform diameter of nearly 31.2 nm. However, after the SiO_2_ coating, the diameter of the nanowires experienced a noticeable increment. The thickness of the SiO_2_ coating was estimated to be around 30.5 nm. Given their long length, the Fe4 nanowires had a tendency to become entangled when in solution. During SiO_2_ coating, Si atoms and OH molecules underwent a reaction to form Si–OH bonds; thus, achieving the same level of separation for Fe4@SiO_2_ nanowires as spherical particles proved to be quite challenging.

Besides, an analysis of FT-IR spectrum was performed on Fe4@SiO_2_ multilayer nanowires, with the results shown in Fig. 4b. It can be seen that the strong absorption peaks located at 1230 cm^−1^ and 1070 cm^−1^ in the spectrum resulted from the asymmetric stretching vibration of Si–O–Si. Moreover, the absorption peaks observed at 974 cm^−1^ and 805 cm^−1^ were attributed to the O–Si (H) and Si–O–Si stretching vibrations, respectively. The vibration peak belonging to the O–Si–O group was located at 454 cm^−1^. Interestingly, the observed spectrum was completely consistent with the characteristic absorption bands of amorphous SiO_2_, indicating that the nanowires in this study were successfully coated with SiO_2_ [21].

**Fig. 5:**
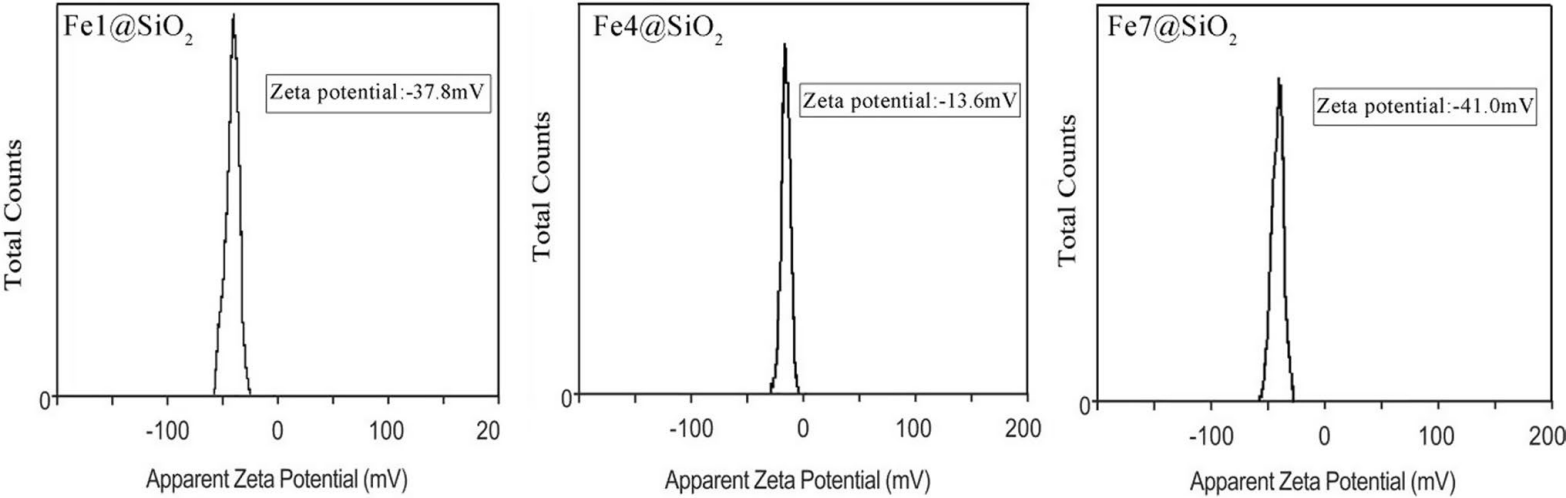
Zeta potential of Fe@SiO_2_ nanowires

To assess the zeta potential, a dispersion of 0.5 mM Fe@SiO_2_ nanowires was prepared, with the relevant results showcased in Fig. 5. Notably, the zeta potentials of the Fe1@SiO_2_ and Fe7@SiO_2_ nanowire dispersions were considerably high with values of −37.8 mV and −41.0 mV, respectively, implying that these coated samples showed good colloidal stability. In contrast, the zeta potential of the Fe4@SiO_2_ nanowires stood at a lower value of −13.6 mV, signifying that the dispersion stability of these nanowires was moderate. This phenomenon occurred because the lengthy Fe4 nanowires had a tendency to interact with one another within the dispersion, making them prone to agglomeration when coated with SiO_2_. In addition, longer Fe nanowires had a greater mass, which can lead to faster sedimentation.

### Relaxation of Fe nanowires

**Fig. 6:**
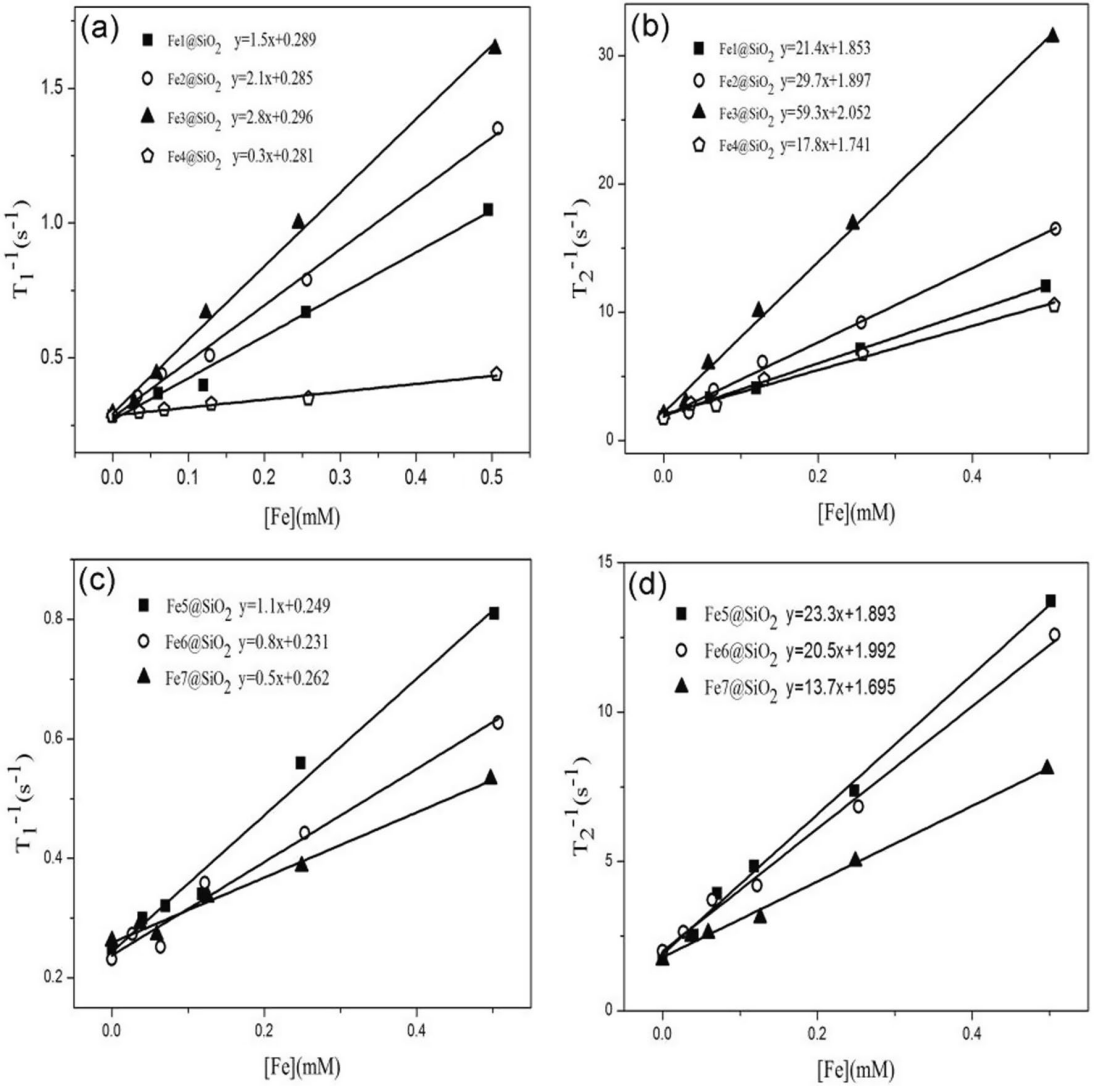
Relaxation properties of Fe@SiO_2_ nanowires **a** 1#–4# obtained a correlation line between 1/T_1_ and Fe concentration in a 1.5 T imager, and the slope of the fitting line was r_1_; **b** 1#–4# obtained a correlation line between 1/T_2_ and Fe concentration in a 1.5 T imager, and the slope of the fitting line was r_2_; **c** 5#–7# obtained a correlation line between 1/T_1_ and Fe concentration in a 1.5 T imager, and the slope of the fitting line was r_1_; **d** 5#–7# obtained a correlation line between 1/T_2_ and Fe concentration in a 1.5 T imager, and the slope of the fitting line was r_2_

The longitudinal relaxation rate (1/T_1_) and transverse relaxation rate (1/T_2_) were computed for the Fe@SiO_2_ nanowires, each having different diameters in the range of 78.1–163.3 nm and lengths in the range of 150–1510 nm. The r_1_ and r_2_ values of these nanowires were obtained from the slope of the linear plot depicted in Fig. 6. The corresponding results are summarized in Table 6.

**Table 6:** Relaxation of Fe@SiO_2_ nanowires

Fe@SiO_2_	Length (nm)	Diameter (nm)	r_1_ (mM^−1^ s^−1^)	r_2_ (mM^−1^ s^−1^)	r_2_/r_1_
Fe1@SiO_2_	150	78.1	1.5	21.4	14.3
Fe2@SiO_2_	270	90.8	2.1	33.7	16.0
Fe3@SiO_2_	620	88.9	2.8	59.3	21.2
Fe4@SiO_2_	1510	95.5	0.3	17.8	59.3
Fe5@SiO_2_	570	105.3	1.1	23.3	21.2
Fe6@SiO_2_	640	139.4	0.8	20.5	25.6
Fe7@SiO_2_	540	163.3	0.5	13.7	27.4

With a constant diameter of the nanowires (around 90 nm), an increase in the length of the Fe@SiO_2_ nanowires led to a gradual rise in their r_1_ value. This increase was ascribed to the elongation of the turnover time (τ_R_) of the nanowires as they became longer, even though their specific surface area (S/V) remained unchanged; thus, the r_1_ value became larger. Additionally, as the length of the nanowire increased to 1510 nm, the r_1_ value of the Fe@SiO_2_ nanowires exhibited a significant decline. This happened due to the extended length of the nanowires, which made them likely to interact with each other in the dispersion, leading to agglomeration. As a result, the probability of the nanowires coming into contact with the surrounding water protons diminished, which in turn caused a decrease in the r_1_ value.

When the length of the nanowire remained unchanged (nearly 500 nm), a reduction in the diameter of the Fe@SiO_2_ nanowires from 163.3 nm to 88.9 nm led to a gradual enhancement in their r_1_ value. This trend likely occurred because as the diameter of the Fe@SiO_2_ nanowires decreased, their S/V value progressively increased. Consequently, there was a higher chance of the nanowires coming in contact with surrounding water protons despite having a shorter τ_R_. In essence, it was found that thinner Fe@SiO_2_ nanowires demonstrated greater r_1_ values.

The r_2_/r_1_ ratios of the Fe@SiO_2_ nanowires are listed in Table 6. It should be mentioned that the r_2_/r_1_ ratios of these nanowires were observed to be greater than 2. Specifically, Fe@SiO_2_ nanowires with r_2_/r_1_ > 10 were used as T_2_-weighted CAs, whereas those with r_2_/r_1_ < 2 were utilized as T_1_-weighted CAs. In general, MRI CAs shorten both T_1_ and T_2_; however, T_1_ CAs enhance both r_1_ and r_2_ values approximately to the same magnitude. While T_1_ CAs amplify the signal intensity of T_1_-weighted MR images, T_2_ CAs do the opposite by diminishing the signal intensity of T_2_-weighted MR images. If the impact of T_2_ contrast effects is too strong, it can considerably decrease the signal intensity of T_1_-weighted MRI. Consequently, the Fe@SiO_2_ nanowires are not as effective as T_1_ CAs. Further optimization is required to make them competitive as T_1_ CAs.

When the nanowire diameter remained unchanged, approximately 90 nm, an increase in the length of the Fe@SiO_2_ nanowires from 150 to 620 nm resulted in a slow rise in their r_2_ value. A similar trend was noticed when the nanowire length was kept constant, around 600 nm, while the diameter of the nanowire progressively decreased from 163.3 nm to 88.9 nm. For slender nanowires, the influence of magnetic crystal anisotropy is minimal and can often be ignored. In these scenarios, shape anisotropy plays a dominant role, leading the magnetization axis of the nanowire to align parallel to its long axis direction [22]. Therefore, when the diameter of the nanowire was kept constant, an increase in the length of the nanowire (Fe1@SiO_2_ → Fe3@SiO_2_) led to a higher aspect ratio of the nanowire. Similarly, when the length of the nanowire remained unchanged, a decrease in the diameter of the nanowire (Fe7@SiO_2_ → Fe5@SiO_2_, Fe3@SiO_2_) resulted in a greater aspect ratio of the nanowire. A higher aspect ratio of the Fe@SiO_2_ nanowires caused an expanded coverage area of the generated magnetic field, which also amplified the effective surface area available for the diffusion of water molecules. Hence, a larger, non-uniform magnetic field was readily formed, promoting the dephasing of adjacent hydrogen protons, which, in turn, reduced the signal intensity of T_2_-weighted imaging. Despite having a higher aspect ratio, Fe4@SiO_2_ nanowires were susceptible to aggregation and sedimentation, which negatively affected their dispersibility, resulting in a decrease in r_2_ value. Hence, it was observed that the longer the Fe@SiO_2_ nanowires and the smaller their diameter, the larger the r_2_ value. Notably, the Fe3@SiO_2_ nanowires exhibited the highest r_2_ value, measuring at 59.3 mM^−1^ s^−1^.

Meanwhile, the r_2_ value of the Fe3@SiO_2_ nanowires was compared against that of Fe_3_O_4_ nanoparticles, as outlined in Table 7. Interestingly, the r_2_ value of the Fe3@SiO_2_ nanowires was slightly higher than that of the Combidex, which stood at 53.0 mM^−1^ s^−1^, inferring that the as-fabricated Fe3@SiO_2_ nanowires showed promise as T_2_ CAs.

**Table 7:** Comparison of r_1_ and r_2_ value of T_1_ and T_2_ CAs used in clinical applications

Relaxation	Complex name	Generic name	Composition	r_1_ (mM^−1^ s^−1^)	r_2_ (mM^−1^ s^−1^)	Ref
T_1_	Gd-DTPA	Magnevist^®^	Gd	3.8	–	[23]
	Gd-DOTA	Dotarem^®^	Gd	3.5	–	[23]
	Gd-HPDO3A	Prohance^®^	Gd	3.6	–	[23]
	DAB-Am64	–	Gd	18.4	–	[23]
T_2_	Ferumoxides	Feridex^®^	Fe_3_O_4_, γ-Fe_2_O_3_	–	120	[24]
	Ferucarbotran	Resovist^®^	Fe_3_O_4_	–	186	[24]
	Ferumoxtran	Combidex^®^	Fe_3_O_4_	–	53	[24]
	MnMEIO	–	MnFe_2_O_4_	–	208	[25]
	Au-Fe_3_O_4_	–	Fe_3_O_4_		114	[24]
	Au-FePt	–	FePt		59	[26]

**Fig. 7:**
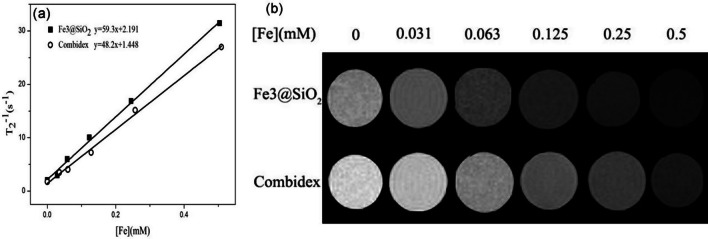
**a** The correlation line between 1/T_2_ and Fe concentration obtained by Fe3@SiO_2_ nanowires and Combidex in 1.5 T imager was r_2_ as the slope of the fitting line; **b** T_2_-weighted MR images of Fe3@SiO_2_ nanowires and Combidex with different Fe concentrations

Under identical conditions, the T_2_ relaxation properties of the Fe3@SiO_2_ nanowires were evaluated in comparison with the commercially available Combidex. As evident from Fig. 7a, the r_2_ value of the Fe3@SiO_2_ nanowires, at 59.3 mM^−1^ s^−1^, was marginally superior to that of Combidex, which measured at 48.2 mM^−1^ s^−1^. Figure 7b displays the T_2_-weighted MR images of the Fe3@SiO_2_ nanowires at varying Fe concentrations alongside the image of the Combidex CAs. With the increase in the Fe concentration, the T_2_-weighted MR images of the Fe3@SiO_2_ nanowires appeared darker, with a more pronounced contrast when compared to the same Fe concentration.

### Cytotoxicity and in vivo toxicity of Fe nanowires

**Fig. 8:**
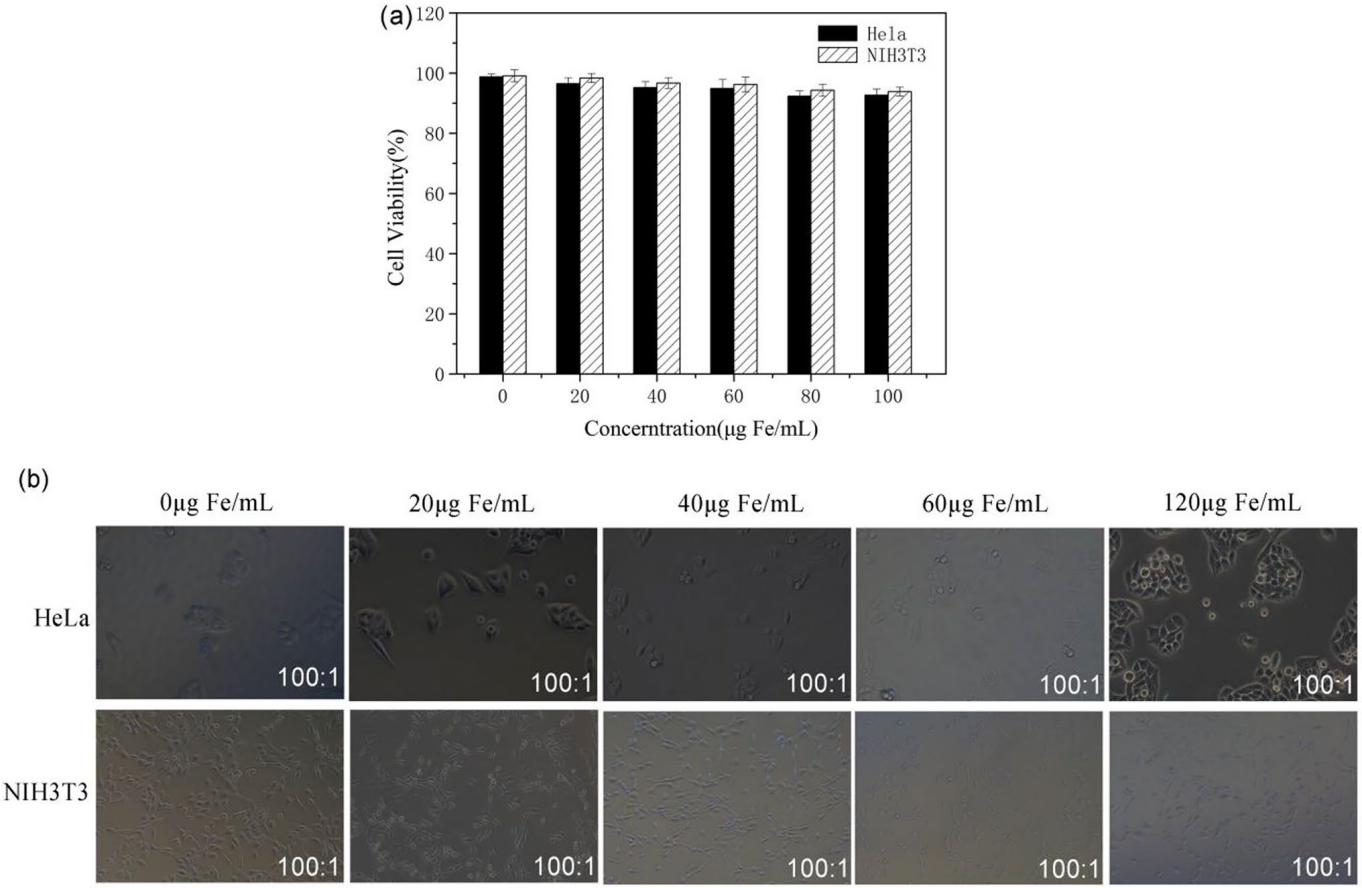
**a** The effects of different concentrations of Fe3@SiO_2_ nanowires on the survival rate of NIH3T3 and HeLa cells after 24 h were detected by CCK8 method; cell morphology of **b** control group and incubated with different concentrations of Fe3@SiO_2_ nanowires for 24 h

Besides, the viability impact of Fe3@SiO_2_ nanowires on cells was assessed using NIH3T3 and HeLa cells as test subjects. The classical CCK8 method was employed to detect changes in cell viability. NIH3T3 and HeLa cells were co-cultured separately with Fe3@SiO_2_ nanowires at different concentrations ranging from 0 to 120 µg Fe/mL for a duration of 24 h. The corresponding outcomes are illustrated in Fig. 8a. It was observed that the survival rates of NIH3T3 and HeLa cells slightly decreased with the increase in Fe3@SiO_2_ nanowire concentration. However, in most instances, the cell survival rate remained high, exceeding 85%. The morphological changes of NIH3T3 and HeLa cells are presented in Fig. 8b. Noticeably, there was not any significant change in the size and morphology of NIH3T3 and HeLa cells when compared to the control group. This suggested that at a concentration of 120 µg Fe/mL, the Fe3@SiO_2_ nanowires demonstrated minimal cytotoxicity.

**Fig. 9:**
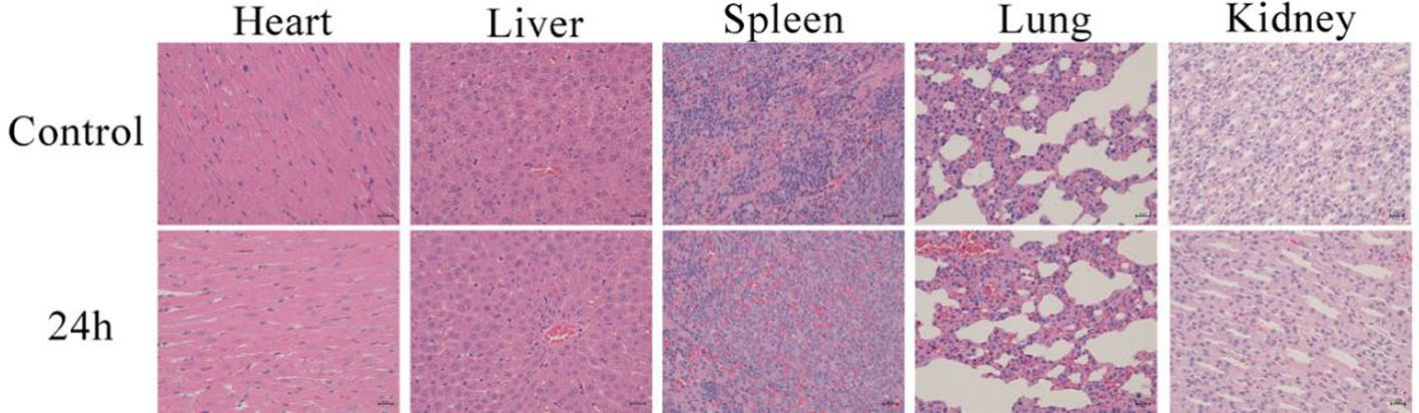
H&E staining images of major internal organs (heart, liver, spleen, lung, and kidney) in nude SD mice (below) and untreated SD nude mice (above) 24 h after injection of Fe3@SiO_2_ nanowires through the tail vein (8.0 mg Fe/kg rat body weight)

To further evaluate the biocompatibility of the Fe3@SiO_2_ nanowires, biopsies were performed. SD rats served as the research objects for this study. For this purpose, they were injected with Fe3@SiO_2_ nanowires with a concentration of 8 mg of Fe/kg of rat weight via the caudal vein. Following a 24 h period, major visceral organs-heart, liver, spleen, lung, and kidney were extracted, processed into paraffin sections, and subsequently stained with H&E. In the visceral slices of each group, the myocardial cells exhibited a short columnar structure without any noticeable lesions. Moreover, there were no signs of fibrosis or vacuoles in the liver and other examined organs, as shown in Fig. 9. This indicated that within the chosen dosage and exposure duration, the Fe3@SiO_2_ nanowires demonstrated low toxicity. Therefore, it can be deduced that the Fe3@SiO_2_ nanowires exhibited excellent biocompatibility.

### In vivo imaging of Fe nanowires

**Fig. 10:**
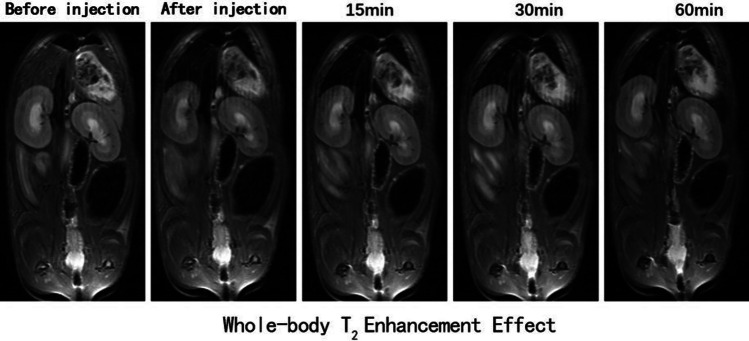
Weighted diagram of T_2_ in coronal position of rats after Fe3@SiO_2_ nanowires (8.0 mg Fe/kg rat body weight) were injected into the lower tail vein at 1.5 T

**Fig. 11:**
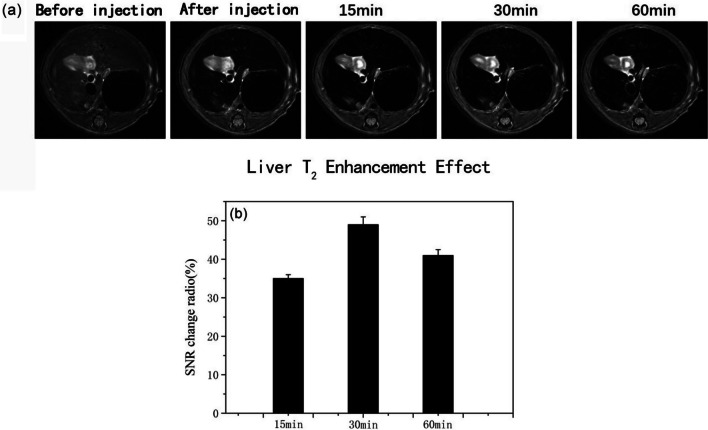
**a** T_2_-weights MR images of liver in SD rats before and after 1.5 T injection of Fe3@SiO_2_ nanowires (8.0 mg Fe/kg rat body weight) at 15, 30, and 60 min (transverse position); **b** analysis of SNR change in transverse T_2_-weight MR images (n = 5)

Meanwhile, the in vivo T_2_ contrast effect of the Fe3@SiO_2_ nanowires was evaluated using healthy SD rats as test subjects. The MRI experiments were conducted on a 1.5 T magnetic resonance scanning system. Initial investigations focused on the whole-body enhancement effect in the SD rats. The results revealed a notable enhancement in the T_2_-weighted MR images of the liver following the administration of Fe3@SiO_2_ nanowires (Fig. 10). Therefore, the liver was selected as the region of interest for the SD rats. In addition, cross-sectional T_2_-weighted MR images of the rat liver were acquired both before and at various intervals (0, 15, 30, and 60 min) after the introduction of Fe3@SiO_2_ nanowires at a concentration of 8 mg Fe/mL (Fig. 11a). Due to challenges in maintaining consistent rat posture before and after the injection, there were slight differences in the weighted images obtained pre and post-injection.

It can be noticed from Fig. 11b that the T_2_-weighted MR images of the cross section of the rat liver exhibited a diminishing signal after 15 min of Fe3@SiO_2_ nanowire injection, with a signal to noise (△SNR = |SNR_post_ − SNR_pre_|/SNR_pre_) of 35% at this time point. The most significant negative contrast enhancement effect was observed after 30 min of injection. The signal attenuation effect in the T_2_-weighted MR images in the transverse section became more pronounced, with an increase in the SNR value by roughly 49%. These results indicated that the synthesized Fe3@SiO_2_ nanowires showed great potential as T_2_ CAs.

## Conclusion

Fe nanowires with varying diameters and lengths were successfully prepared using the constant piezoelectric deposition method. These nanowires were thoroughly characterized for their morphology, composition, and structure. Additionally, the study assessed the relaxation properties, cytotoxicity, and biocompatibility of SiO_2_-coated nanowires. Their in vivo imaging effects were also investigated. The main findings of this experiment are presented below.

1. The Fe@SiO_2_ nanowires acted as excellent T_2_ CAs. As the length of the nanowires increased and their diameter decreased, there was a corresponding rise in the r_2_ value. Among all the prepared nanowires, the Fe3@SiO_2_ nanowires exhibited the maximum r_2_ value, recorded at 59.3 mM^−1^ s^−1^.
2. When exposed to specific concentrations of the synthesized Fe@SiO_2_ nanowire (0–120 µg Fe/mL) for a duration of 24 h, NIH3T3 and HeLa cells experienced almost no toxicity. As a result, nanowires manifested commendable biocompatibility after a tail vein injection with a dose of 8.0 mg of Fe/kg of rat body weight.
3. Moreover, a negative enhancement effect was noticed in the liver of SD rats during small animal imaging at 1.5 T. This suggested that the as-prepared Fe nanowires have the potential to serve as T_2_ MR CAs, with the possibility of tuning their performance by adjusting their size.

## Outlook

1. We successfully developed a one-dimensional Fe nanowire CAs that showcases effective liver imaging effects and exhibits good biocompatibility. Moreover, the synthesis approach is simple and easy, paving the way for scalable production. We believe that in the future, there is potential for further optimization of the structure and composition of CAs to improve their imaging performance. In addition, owing to the large specific surface area of Fe nanowires, they can be readily coated with functional molecules, allowing for the attachment of other biological molecules to target the nanowires toward specific cell types. Linear or rod-shaped one-dimensional magnetic nanostructures offer advantages such as extended blood circulation times and superior transport and penetration characteristics in porous tissues like tumor sites. In our subsequent research, we aim to investigate the in vivo circulation ability of Fe nanowires and conduct a comprehensive assessment of their safety.
2. Furthermore, our observations revealed that the peak negative contrast enhancement effect occurred approximately 30 min post-injection. As time progressed, at 180 min after injection, the SNR at the transverse position in the T_2_-weighted images slightly decreased, indicating that the optimal in vivo T_2_ imaging effect of the Fe@SiO_2_ nanowire was achieved within the first 30 min. Therefore, our focus was limited to analyzing the histology of some selected organs in mice 24 h post-injection to demonstrate the biocompatibility of the synthesized Fe@SiO_2_ nanowires. However, the specific mechanisms governing the accumulation and distribution of the Fe@SiO_2_ nanowires across various organs remain ambiguous. Equally uncertain is the ability of the body to excrete these nanowires and primary metabolic pathways. Further investigations are imperative to elucidate these aspects.

## Data Availability

The data that support the findings of this study are available from the corresponding author, [Liyuan Gu], upon reasonable request.
